# Operationalizing fear of cancer recurrence care: a modified Delphi study establishing a consensus-based clinical pathway for Danish cancer survivors

**DOI:** 10.2340/1651-226X.2026.46444

**Published:** 2026-09-24

**Authors:** Johanne Dam Lyhne, Pernille Bech, Eva Rames Nissen, Christina Maar Andersen, Anne Katrine Hartmann Søby, Brigitta R Villumsen, Henriette Vind Thaysen, Laura Sandholdt Jensen, Iben Husted Nielsen, Thea Otto Mattsson, Anne Johansen, Emma Skov Rahbæk, Ulrike Johansen, Frederik Lynge Overgaard, Lise Ventzel, Lars Henrik Jensen, Matteo Allegretti, Eriseld Krasniqi, Allan ’Ben’ Smith, Anders Bonde Jensen

**Affiliations:** a Department of Oncology, University Hospital of Southern Denmark, Vejle, Denmark; b Department of Regional Health Research, University Hospital of Southern Denmark, Odense, Denmark; c Department of Clinical Medicine, Aarhus University, Aarhus, Denmark; d Department of Oncology, Aarhus University Hospital, Aarhus, Denmark; e Unit for Psychooncology and Health Psychology, Aarhus University and Aarhus University Hospital, Aarhus, Denmark; f REHPA, The Danish Knowledge Centre of Rehabilitation and Palliative Care, Odense University Hospital, Nyborg, Denmark; g Department of Anaesthesiology and Intensive Care, Gødstrup Hospital, Herning, Denmark; h Department of Surgery, Aarhus University Hospital, Aarhus, Denmark; i Clinic for Complex Late Effects After Cancer, Department of Social Medicine, Aalborg University Hospital, Aalborg, Denmark; j Department of Hematology, Copenhagen University Hospital, Rigshospitalet, Copenhagen, Denmark; k Late Effect Clinic, Department of Clinical Oncology, Odense University Hospital, Odense, Denmark; l Cancer Rehabilitation, Vejle Municipality, Vejle, Denmark; m Department of Cardiothoracic Surgery, Rigshospitalet, Copenhagen University Hospital, Copenhagen, Denmark; n Translational Oncology Research Unit, Department of Research and Advanced Technologies, IRCCS Regina Elena National Cancer Institute, Rome, Italy; o Phase IV Clinical Studies Unit, IRRCS, Regina Elena National Cancer Institute, Rome, Italy; p The Daffodil Centre, The University of Sydney, and Cancer Council NSW, Sydney, Australia

**Keywords:** Cancer survivors, neoplasms, clinical pathway, Delphi method, practice guideline, psycho-oncology

## Abstract

**Background and purpose:**

Fear of cancer recurrence (FCR) is a prevalent concern among cancer survivors. Although evidence-based FCR guidelines exist, their implementation requires further operationalization. This study aimed to establish expert consensus on a clinical pathway to operationalize guideline-concordant FCR care in Denmark.

**Patient/material and methods:**

A modified Delphi study was conducted, comprising Round 0 and three Delphi rounds. In Round 1, 112 experts were invited, and 80 participated (71%) in an online survey rating proposed pathway statements. Twenty-seven participants attended a consensus workshop (Round 2) to discuss and refine statements not reaching consensus in Round 1. In Round 3, 71 participants (63%) re-rated the revised statements from Round 2. Consensus was defined as ≥ 80% agreement.

**Results:**

Consensus was achieved for 10 statements in Round 1 and 6 revised or newly formulated statements in Round 3, including systematic screening of all cancer survivors with validated tools using a two-step approach, and initiation of screening at transition from treatment to follow-up. Consensus was achieved for need-based assessment guided by symptom severity and patient-expressed need. A matched-care model, aligning treatment intensity with FCR severity, achieved consensus in Round 1. Ongoing monitoring through re-screening during follow-up and post-intervention was endorsed. Timing of assessment and the management of FCR treatment non-response did not reach consensus.

**Interpretation:**

This Delphi study provides a consensus-based clinical pathway for FCR, including systematic screening, needs-based assessment, and matched-care allocation. The findings address key implementation gaps in existing FCR guidelines and offer a structured framework for integrating FCR care into routine practice.

## Introduction

Fear of cancer recurrence (FCR), defined as the ‘*fear, worry or concern that the cancer will come back or progress*,’ [1] is one of the most common and persistent late effects among adult cancer survivors living beyond early-stage cancer [2, 3]. While many survivors experience transient or manageable fears, up to three in five cancer survivors report moderate or higher levels of FCR, while approximately one in five reports severe FCR at a level considered clinically significant, which is associated with reduced quality of life, increased psychological distress, and higher healthcare utilization [4–6]. FCR is closely intertwined with physical symptoms, which may trigger or exacerbate fears of recurrence and complicate the interpretation of symptoms during survivorship [5]. FCR has also been associated with other late effects, including sleep disturbances and anxiety, further highlighting its interconnectedness with broader survivorship concerns [7]. As survival rates continue to improve, awareness of the late effects of cancer and its treatment has increased, making the psychosocial consequences of cancer, including FCR, an increasingly important component of survivorship care [8].

Over the past decade, a growing body of research has demonstrated that FCR can be identified and effectively treated. Brief screening tools [9–12] have been developed to facilitate early identification, and in a meta-analysis, several psychological interventions – particularly cognitive and metacognitive approaches – have shown efficacy in reducing FCR severity [13]. Despite this, FCR management, including the clinical identification, assessment, and the management of FCR as part of survivorship care, is not systematically implemented in routine clinical practice.

The first clinical guideline to support healthcare professionals in identifying and managing FCR was published in 2024 [14]. In Denmark, a national clinical guideline for FCR in adult cancer-free survivors following treatment with curative intent has recently been published, providing evidence-based recommendations on screening and treatment [15]. These guidelines provide recommendations regarding what should optimally be done but offer limited guidance on how to operationalize them within the existing healthcare systems.

Translating guideline recommendations into clinical practice requires decisions regarding workflow, roles and responsibilities, timing of key activities, and integration across cross-sectoral settings (e.g. primary and tertiary care). These aspects are often context-dependent and cannot be addressed by empirical evidence alone. To support implementation, structured methods are needed to integrate available evidence with clinical expertise and expertise by experience, thereby developing a feasible and acceptable clinical pathway that provides operational and context-specific guidance. In this context, the purpose of this present study was not to develop new clinical recommendations but to operationalize existing guideline recommendations into a clinically applicable pathway for routine care. Such an approach has previously been used to develop a consensus-based clinical pathway for FCR in Australia [16].

The Delphi method [17] is a structured and iterative consensus process that systematically integrates expert judgment through repeated rounds of anonymous rating, feedback, and refinement. The Delphi method is widely used to establish consensus in areas where evidence is limited. By combining iterative rounds of ratings with feedback and discussion, Delphi studies allow for the systematic refinement of complex clinical and organizational questions. Previous Delphi studies have been used to develop clinical pathways for psycho-oncological symptoms, including FCR, in other healthcare settings, demonstrating the feasibility of this approach [16, 18].

The aim of the present study was to establish an expert-based national consensus on the practical operationalization of the Danish clinical guideline for FCR in cancer-free survivors. Using a modified Delphi design, this study aimed to determine how existing evidence-based recommendations for addressing FCR can be translated into a structured and clinically applicable pathway within the Danish healthcare system.

## Patients/material and methods

### Ethics

This study was conducted in accordance with the Conducting and Reporting Delphi Studies (CREDES) recommendations [19]. Ethical approval for the study was obtained from the University of Southern Denmark Research Ethics Committee (No. 695). All participants received written study information and provided informed consent electronically before participation.

### Participants and recruitment

Eligible participants were adult healthcare professionals, researchers, and patient representatives with relevant experience or expertise in cancer survivorship and/or FCR. Participants were recruited nationally from the members list of the Danish Psycho-Oncology Cooperative group, consisting of physicians, psychologists, nurses, researchers, and patient representatives. Clinicians working in Danish late-effects clinics, including oncologists, nurses, psychologists, and allied health professionals, were also invited to participate. A total of 112 potential participants were invited by email. All invitees were eligible to participate in each Delphi round regardless of participation in previous rounds, and no additional participants were recruited during the study.

### Study design

**Figure 1:**
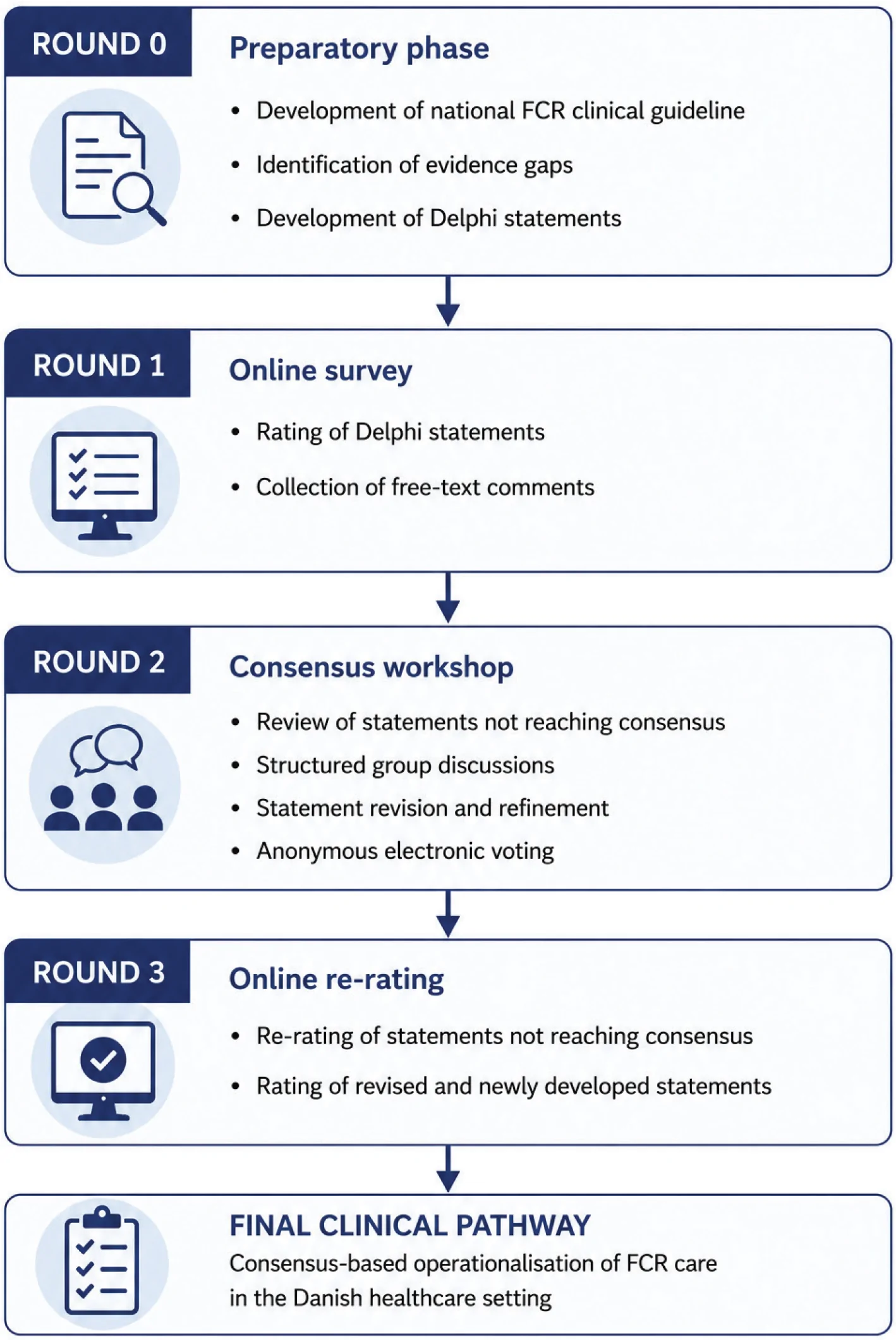
Study design.

This study consisted of four phases: An initial preparatory phase (Round 0), followed by three Delphi rounds, see Figure 1. The design was modified by incorporating an in-person statement-refinement workshop (Round 2) between the two survey-based Delphi rounds. All survey rounds and the in-person workshop were conducted in Danish.

Participants received an online information sheet, consent form, and demographic questionnaire before rating Delphi statements grouped by pathway components. Participants were asked to indicate their level of agreement with each statement as representing an optimal component of an FCR clinical pathway. Responses were recorded on a five-point Likert scale ranging from ‘strongly disagree’, ‘disagree’, ‘neutral’, ‘agree’, and ‘strongly agree’. Indicating ‘not my field of expertise’ was an option for all statements.

Each section included the relevant guideline recommendation and supporting evidence and identified research gaps. Non-responders received one reminder after 2 weeks, and the online surveys for Rounds 1 and 3 were each open for 3 weeks, separated by a 1-week interval during which the Round 2 workshop was conducted. No additional incentives were provided.

### Consensus definition and data analysis

Consensus was defined a priori as ≥ 80% agreement (‘agree’ or ‘strongly agree’), excluding responses marked as ‘not within my area of expertise’. Statements meeting this threshold were included in the final clinical pathway. As responses were required for all statements, there was no missing data.

Agreement proportions were calculated for each statement in each round. Statements with 50–79% agreement were revised based on qualitative feedback; those with < 50% agreement were removed. Where appropriate, statements were rephrased into alternative versions for reevaluation.

#### Round 0

Round 0 consisted of developing a national evidence-based clinical guideline for FCR, informed by systematic literature reviews and conducted according to Danish Health Authority methodology [15–22].

The guideline identified evidence-based recommendations for screening and treatment. In this context, screening refers to the systematic identification of cancer survivors who may experience elevated levels of FCR using brief validated instruments, whereas assessment refers to a more comprehensive evaluation of the nature, severity, and consequences of FCR to guide treatment decisions. However, evidence was limited regarding key aspects of clinical implementation, particularly the organization and delivery of care, including screening timing, responsibility for assessment, and structuring of treatment pathways.

These gaps formed the basis for the Delphi process, which aimed to develop consensus-based recommendations for practical guideline implementation in the Danish healthcare setting.

Twenty-four statements were developed by the research group based on the recommendations and identified evidence gaps in the Danish clinical guideline for FCR, with the statement-development process inspired by a similar Delphi process conducted in Australia in 2024 [16]. Statements assigning responsibility to specific professional groups were excluded because role allocation is context-dependent and determined locally according to available resources.

#### Round 1

Round 1 was conducted as an online survey using REDCap [23, 24] (University of Southern Denmark). Participants rated each statement and provided free-text comments suggesting modifications.

#### Round 2

Round 2 consisted of an in-person workshop. All 112 participants were invited to participate. The workshop focused exclusively on statements that had not reached consensus in Round 1.

Participants were presented with anonymized summary data from Round 1, including quantitative ratings and free-text comments. They then engaged in structured, non-anonymous group discussions to explore sources of disagreement and refine statement wording. Participants were divided into four subgroups, each reviewing statements related to a specific pathway component. Revised or newly developed statements were presented to the entire group and subjected to anonymous electronic voting. The purpose of the voting was to assess satisfaction with the revised or newly developed statements, rather than to establish consensus. All statements were subsequently carried forward to Round 3.

#### Round 3

Round 3 was conducted as a second online survey. Participants were asked to re-rate the original statements that had not reached consensus in Round 1, together with revised and newly formulated statements developed during the Round 2 workshop.

In addition to rating the statements, participants were in five cases asked to indicate their preferred formulation among multiple alternative versions of the statements.

## Results

### Participants

Of the 112 individuals invited, 80 completed Round 1 (71%) (March 2026). A total of 27 participants attended the Round 2 workshop (24%) (March 2026). In Round 3, 71 participants completed the survey (63%) (April 2026). Participants represented a multidisciplinary group of clinicians, researchers, and patient representatives from across Denmark. Detailed participant characteristics are presented in Table 1. Participant characteristics were collected at baseline and are therefore reported for Round 1 respondents.

**Table 1:** Participant characteristics.

Characteristic	*N* (%) of respondents (*n* = 80)
**Age**	
21–30	2 (2%)
31–40	19 (24%)
41–50	26 (33%)
51–60	24 (30%)
61–70	7 (9%)
71–80	2 (2%)
**Gender**	
Male	14 (18%)
Female	66 (82%)
**Primary role**	
Researcher	35 (44%)
Clinician	39 (49%)
Leadership	3 (4%)
Patient representative	3 (4%)
**Primary focus if clinician**	
Psychology	16 (41%)
Nursing	9 (23%)
Oncology	11 (28%)
Palliation	1 (3%)
Other	2 (5%)
Clinical unit*	
University hospital	38 (56%)
Regional hospital	14 (21%)
Municipality	2 (3%)
Danish Cancer Society	6 (9%)
Late effect clinic	9 (14%)
Private clinic	3 (5%)
Other	4 (8%)
**Years worked with cancer survivors**	
0–2	5 (6%)
3–5	16 (20%)
6–10	21 (26%)
10+	24 (30%)
Not relevant	14 (18%)
Role caring for cancer survivors with FCR if clinician*	
Screening and/or assessment	19 (27%)
Referral to psychosocial support	28 (42%)
Provision of psychosocial support	9 (12%)

FCR: fear of cancer recurrence.

* Respondents could select multiple responses.

#### Round 1

In Round 1, consensus was achieved for 10 of 24 (42%) statements in the proposed FCR clinical pathway, see Table 2. For the full list of results of all rounds and exact formulation of statements, see Supplementary File 2. The number of participants reporting ‘not my field of expertise’ ranged from 0 to 6.

**Table 2:** Statements achieving consensus on Round 1 and Round 3.

Domain	Statement	Agreement (%)	Round achieved
Screening	All cancer survivors should be systematically screened for FCR	81	Round 1
	A brief, validated tool, administered either in written or oral form, should be used to identify cancer survivors with possible FCR	89	Round 1
	A two-step screening approach should be used: (1) a brief (e.g. single-item) screening tool to identify individuals with possible FCR; (2) a longer (e.g. 9-item) tool to assess severity among those scoring above the cut-off	91	Round 1
	Screening for FCR should be conducted prior to a cancer survivor’s follow-up consultation to support discussion during the consultation	82	Round 1
	Screening for FCR should be initiated at the transition from treatment (e.g. surgery, systemic therapy or radiotherapy) to follow-up	83	Round 3
	Results from FCR screening should be included in communication to the general practitioner	99	Round 3
	Screening for FCR should be conducted as part of follow-up consultations in general practice	91	Round 3
	Clear workflows for FCR screening should be established, and relevant personnel should be trained to conduct screening, interpret results, and determine indication for assessment	97	Round 3
Assessment	Training is needed to support healthcare professionals in conducting FCR assessment	92	Round 1
	Assessment should take place if the patient reports moderate to severe FCR on screening or expresses a need (verbally or non-verbally) for assessment	94	Round 3
	When screening and assessment results indicate severe FCR, the cancer survivor should be evaluated by a psychologist or psychiatrist to confirm the need for specialized FCR treatment	81	Round 1
Treatment	Cancer survivors should be offered an intervention at the treatment level (1, 2, or 3) that matches the severity of their FCR (matched-care)	81	Round 1
	Treatment offered to cancer survivors with FCR should take into account FCR severity as well as patient preferences and ability to engage in self-help versus clinician-delivered interventions	95	Round 1
Follow-up	Re-screening for FCR during routine follow-up consultations is an appropriate way to monitor changes and guide further assessment and treatment if needed	82	Round 1
	Patients should be re-screened 6 months after intervention to assess long-term effects	82	Round 1
	If the cancer survivor does not improve following the offered FCR intervention, or continues to report treatment-requiring FCR at re-screening, care should be reevaluated in collaboration with the cancer survivor to determine further treatment	99	Round 3

FCR: fear of cancer recurrence.

Regarding screening, 4/9 statements reached consensus. There was consensus that all cancer survivors should be systematically screened for FCR, and that screening should be conducted prior to follow-up consultations. Participants further endorsed the use of validated screening tools and a two-step screening approach. While initiation of screening at the time of diagnosis was strongly rejected (70% disagreement), screening at the completion of hospital-based treatment received the highest level of agreement (71%) among the proposed screening initiation timepoints. Similarly, no consensus was reached regarding responsibility for reviewing and responding to screening results, although training all clinical staff to conduct FCR screening and respond appropriately received 66% level of agreement.

Regarding assessment of patients with potential FCR, 2/7 statements reached consensus, including the need for professional training to enable conversations about FCR with cancer survivors and for referral to specialized mental health services for patients with severe FCR. However, consensus was not reached regarding which patients should undergo assessment or the timing of assessment.

In the treatment and follow-up domain, 4/8 statements reached consensus. Participants endorsed a matched-care model, whereby intervention intensity is aligned with FCR severity and supported incorporating patient preferences into treatment decisions. In contrast, a stepped-care model did not achieve consensus (36% agreement; 44% disagreement). Consensus was achieved regarding the use of re-screening during follow-up and re-screening 6 months after intervention. Statements related to management of the non-responders did not reach consensus.

In total, four statements received less than 50% agreement and were excluded after Round 1, see Supplementary File 1.

#### Round 2

The Round 2 workshop focused on revising the 10 statements that reached 50–79% agreement in Round 1. Following group discussion, two statements were reformulated, while two others were split into separate statements to better reflect nuances identified during the workshop. In two instances, three related statements were consolidated into a single revised statement, resulting in one revised statement concerning assessment and one concerning follow-up. In addition, two new statements addressing the role of general practice in FCR management were developed.

Several revised statements received strong support during the anonymous workshop voting. For example, initiating screening at transition from treatment to follow-up was endorsed by 85% of workshop participants, and integrating screening results into communication with general practitioners was endorsed by 93%.

In total, 10 refined or new statements were carried forward to Round 3 for reevaluation.

#### Round 3

Round 3 included the 10 original statements that had not reached consensus in Round 1, together with 10 revised or newly developed statements arising from the Round 2 workshop. None of the original statements reached consensus in Round 3. For the revised or new statements, consensus was achieved for initiating screening at the transition from treatment to follow-up, see Table 2. Consensus was also achieved for several organizational elements, including that screening results should be communicated to general practitioners, that screening should be conducted as part of follow-up in general practice, and the need for clear workflows and trained personnel. No consensus was reached regarding whether screening should be repeated immediately following follow-up consultations, with both alternative formulations remaining well below the consensus threshold.

Regarding assessment, consensus was achieved for a formulation specifying that assessment should be conducted when patients report moderate-to-severe FCR or express a need for support either verbally or non-verbally. Consensus was not achieved regarding the timing of assessment, including whether it should be conducted as part of follow-up consultations (77% agreement).

Regarding treatment and follow-up, consensus was achieved for the revised consolidated formulation, stating that care should be individually reevaluated in collaboration with the patient in cases of persistent or treatment-requiring FCR at re-screening. In contrast, other aspects of post-intervention management did not reach consensus. These included offering booster sessions (78% agreement) and continued screening and self-management support for patients with mild FCR (59% agreement).

In total, six statements reached consensus in this round.

### Final clinical pathway

**Figure 2:**
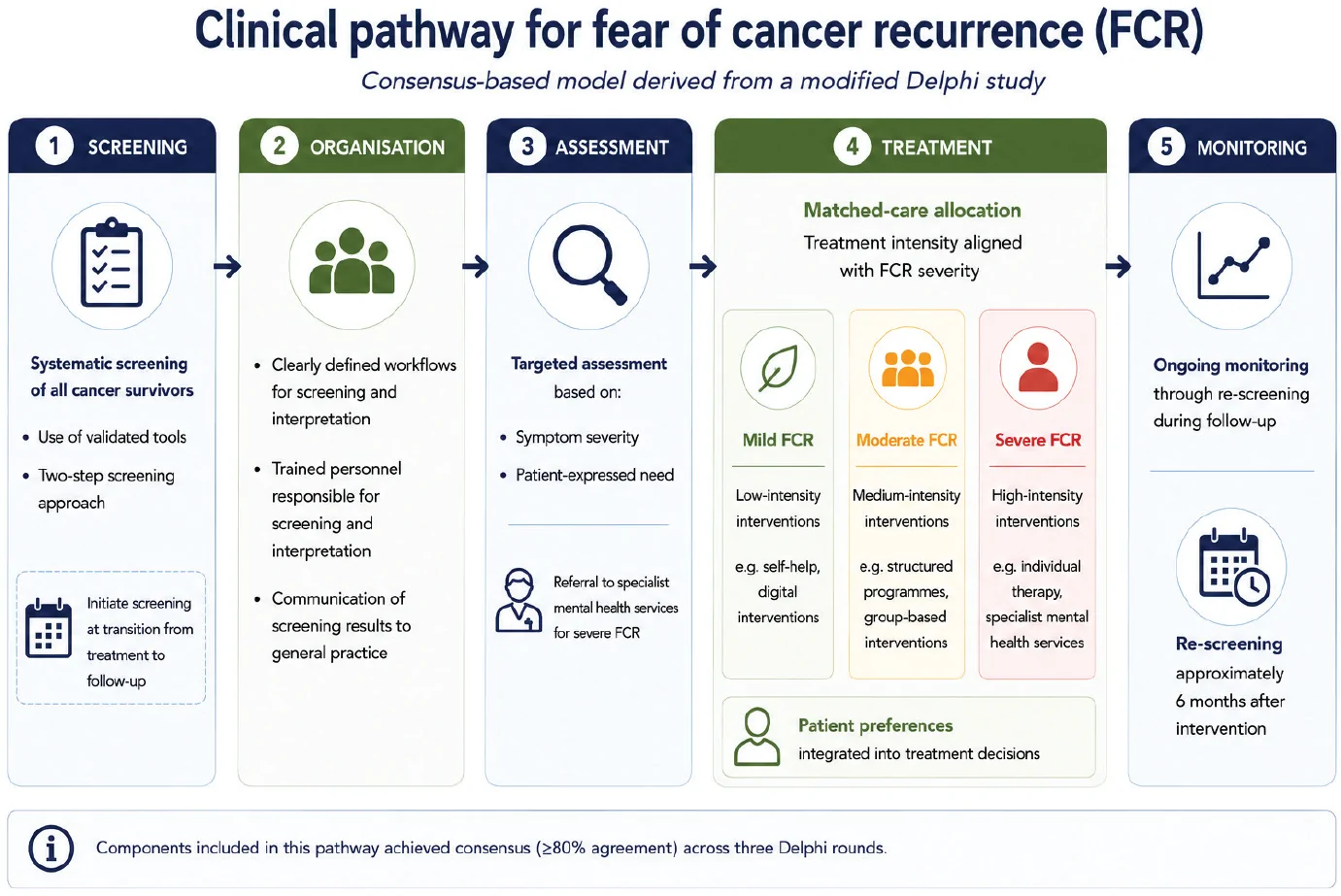
Clinical pathway for fear of cancer recurrence (FCR).

The final clinical pathway was constructed from the 16 statements achieving consensus across the three rounds, see Table 2 and Figure 2.

## Discussion and conclusion

The present study established a national expert consensus on how the Danish clinical guideline for FCR could be operationalized into routine clinical practice. Using a modified Delphi approach, consensus was achieved for several key components of a clinical pathway, including systematic screening using brief validated instruments, structured need-based assessment, allocation to treatment according to a matched-care model, and re-screening during routine follow-up. Consensus was not achieved regarding the optimal timing of FCR assessment. The pathway was developed as a cross-diagnostic framework, with the same core principles intended to apply across cancer types rather than as cancer-specific care pathways. This approach reflects an understanding of FCR as a psychological response to the experience of cancer and its potential recurrence, rather than as a phenomenon specific to a particular cancer diagnosis.

A central finding of this study is the endorsement of systematic screening of all cancer survivors, supported by the use of validated tools and a two-step screening approach. These elements were endorsed in Round 1, suggesting that routine identification of FCR is both conceptually accepted and considered clinically relevant across professional groups. Existing tools, such as the FCR-1r and Fear of Cancer Recurrence Inventory (FCRI) [25], can be used for this purpose. While consensus was ultimately achieved for initiating screening at the transition from treatment to follow-up, the difficulty in reaching consensus highlights ongoing discussion regarding the trajectory of FCR, which includes early transient elevations related to uncertainty around treatment outcomes [16] as well as reluctance of clinicians to address FCR at this timepoint [26]. The consensus-based initiation of screening at the transition from treatment to follow-up is well aligned with the proposed clinical criterion of a minimum duration of approximately 3 months for severe FCR [27], as this timepoint may allow transient fears associated with diagnosis and treatment to subside while facilitating identification of more persistent FCR. In addition, initiating screening at this timepoint may still facilitate early identification of survivors with moderate FCR, allowing timely support and intervention to be offered before worries and their impact on everyday life escalate.

No consensus was reached regarding the timing of assessment within the clinical pathway. The consensus that assessment should also be initiated when patients express a need for support places patient-expressed need alongside symptom severity as a basis for assessment. This patient-centered approach is supported by research, showing that cancer survivors experiencing FCR report diverse support needs, including psychological support and practical guidance [28]. A similar finding was reported in the Australian study [16] and might indicate that while there is agreement on criteria for assessment, there is less clarity regarding when FCR warrants formal assessment and who should be responsible for conducting it in routine care. Conducting assessment as part of follow-up consultations achieved relatively high agreement (77%) but did not meet the predefined threshold for consensus. The lack of clear consensus in this area may reflect ongoing uncertainty regarding the future organization of follow-up care in Denmark, including anticipated changes associated with national cancer initiatives that envisage a greater role for general practice in survivorship care [29].

Another key finding is the clear endorsement of a matched-care model for treatment, as also recommended in the Canadian guideline [14], in which treatment intensity is aligned with the severity of FCR at the time of assessment. This individualized, needs-based approach is conceptually similar to what has recently been described as a *stratified stepped-care model* [30] and is consistent with broader risk- and needs-stratified survivorship care models [31]. In contrast, a traditional stepped-care model, where cancer survivors receive universal care regardless of initial FCR levels and are subsequently stepped up to more intensive interventions if needed, as recommended in the Australian FCR clinical pathway, was excluded. This finding indicates a clear preference for an individualized, needs-based allocation of care, despite stepped-care models being widely advocated in psycho-oncology and cancer survivorship care because of their potential to optimize resource utilization and improve access to psychosocial support [32, 33]. Nevertheless, recent evidence suggests no clear differences in clinical outcomes between stepped-care and matched-care approaches, while comparative evidence regarding their relative cost-effectiveness remains limited [30]. While the feasibility of a matched-care model has been demonstrated in a recent pilot study [34], comparative studies are needed to determine the optimal approach to allocating FCR interventions in clinical practice, including the relative effectiveness, acceptability, and cost-effectiveness of matched-care and stepped-care approaches.

## Strengths and limitations

A key strength of this study is the use of a structured and iterative Delphi process, combining anonymous rating with an in-person statement-refinement workshop. This study also included a multidisciplinary national panel, enhancing the relevance and potential generalizability of the findings within the Danish healthcare context.

Several limitations should be acknowledged. Participation in the workshop was limited to a subset of participants, which may have influenced the refinement of items in Round 2. While the workshop facilitated exploration of nuanced perspectives, social desirability bias, group dynamics, and existing professional hierarchies may have influenced both discussions and statement refinement. Furthermore, the desire to reach agreement may have reduced the expression of dissenting views. Confirmation bias may also have affected both participants and researchers, as preexisting assumptions and professional experiences could have influenced the interpretation and prioritization of viewpoints.

Although response rates were high across rounds, attrition between rounds may have introduced bias. As with all Delphi studies, the findings reflect expert consensus informed by limited empirical evidence and should be interpreted accordingly. Furthermore, this study did not address profession-specific responsibilities within the pathway, and the Delphi panel did not include representatives from the general practice. General practitioners were unfortunately not represented in the initial recruitment pool, which was based on members of the Danish Psycho-Oncology Cooperative Group and clinicians working in late-effects clinics. Moreover, the initial Delphi statements did not address a role for general practice; this emerged during the Round 2 workshop, when two new statements concerning the role of general practice were developed. Consequently, these recommendations reflect endorsement by the participating experts rather than consensus including the views from general practitioners and should be validated with relevant primary care stakeholders before implementation. This highlights the importance of involving primary care stakeholders from the outset when developing cross-sectoral survivorship pathways. The composition of the expert panel may have influenced the findings. Although clinicians and researchers were similarly represented, patient representation was limited to three participants. This may have reduced the diversity of experiential perspectives brought into the consensus process and should be considered when interpreting the findings.

As the national clinical guideline focuses only on cancer-free survivors, the care pathways for patients living with advanced cancer were not included in the present study. Although several statements may be relevant to all cancer survivors, further research is needed to determine relevance of these statements for a patient population living with cancer, where evidence on how to manage fear of recurrence and progression is more limited [35].

### Clinical and implementation implications

Internationally, implementation of the proposed pathway is likely to depend on the degree to which survivorship and late-effects care are embedded in national cancer policy. Although developed within the Danish healthcare system, the present study may have relevance beyond the national context. The process of translating evidence-based recommendations into a clinically applicable pathway may provide a useful model for other healthcare settings seeking to operationalize FCR care. However, implementation of the resulting pathway will need to be adjusted according to local healthcare structures, available resources, professional roles, and existing survivorship services. In healthcare systems where survivorship care has received sustained political and organizational attention, such as Denmark, implementation may be facilitated by existing late-effects services. In contrast, in countries where survivorship care remains less well structurally developed, additional implementation barriers may be expected.

Italy provides an illustrative example. The Italian National Oncology Plan 2023–2027 recognizes long-term survivorship, rehabilitation, tertiary prevention, psychosocial support, quality of life, and return to active life after cancer as important components of cancer care. At the same time, it highlights the need to strengthen integrated, multidisciplinary, and territorially coordinated care for cancer-free survivors [36]. Implementing an FCR pathway in such contexts is likely to require more than the adoption of screening tools. Potential implementation barriers include regional heterogeneity in oncology networks and survivorship pathways, limited formalization of shared hospital-community workflows for survivorship and late-effects care, unclear professional responsibility for FCR assessment, and lack of reimbursement mechanisms. These considerations suggest that implementation should be adapted to the maturity of the healthcare system. In countries with less established survivorship infrastructure, a feasible strategy may be to pilot FCR screening within selected cancer centers or regional oncology networks, define minimum referral and escalation criteria, train oncology nurses and clinicians in FCR assessment and management, and progressively link hospital-based pathways with primary care and community psychosocial services. Thus, the proposed pathway may serve not only as a clinical framework but also as a roadmap for developing survivorship services in healthcare systems where late-effects care is still evolving.

## Conclusions

This modified Delphi study established consensus on key elements required to operationalize a clinical pathway for FCR based on the Danish clinical guideline for FCR. The resulting pathway supports systematic screening, needs-based assessment, and matched-care allocation while identifying areas of uncertainty requiring further research and local adaptation. The findings provide an important step toward translating guideline recommendations into clinical practice and may inform implementation of FCR care across healthcare systems with different levels of survivorship care infrastructure.

## Supplementary Material





## Data Availability

The data that support the findings of this study are available from the corresponding author upon reasonable request.
