# Supplementary material for: Operationalizing fear of cancer recurrence care: a modified Delphi study establishing a consensus-based clinical pathway for Danish cancer survivors
Source: Acta Oncol. 2026 Sep 24;65:46444. doi: 10.2340/1651-226X.2026.46444 (PMC13615413; doi:10.2340/1651-226X.2026.46444)
Supplement: Supplementary file 1 [file AO-65-46444-s1.pdf]

Supplementary material has been published as submitted. It has not been copyedited, or typeset by Acta Oncologica

From guideline to clinical practice: A modified Delphi study on operationalizing fear of cancer recurrence care

Supplementary File 1

This supplementary file summarizes the fear of cancer recurrence (FCR)-specific recommendations from the Danish national clinical guideline on anxiety, depression and fear of cancer recurrence in cancer survivors. Recommendations are presented in English as published in the official guideline.

No.	Domain	Recommendation	Evidence level*
1	Identification	Cancer survivors with symptoms of fear of recurrence should be identified.	D
2	Screening	Brief screenings (1–2 short questions) may be used as an initial method to detect symptoms of fear of recurrence. Subsequently, validated instruments with established cut-off scores (e.g., patient-reported outcome [PRO] questionnaires) should be used.	B
7	Mild FCR	Cancer survivors with normal or mild symptoms of fear of recurrence can be offered information, internet-based self-help programs, or physical exercise programs.	D
8	Moderate FCR	Cancer survivors with moderate symptoms of fear of recurrence may be offered moderately intensive interventions, such as psychoeducational programs, relaxation training, and/or professionally led support groups.	D
9	Severe FCR	Cancer survivors with severe symptoms of fear of recurrence should be offered intensive interventions specifically targeting fear of recurrence, such as cognitive behavioral therapy (CBT) or mind-body interventions.	A

** Evidence levels (A–D) correspond to the grading system used in the Danish national clinical guideline, based on the Oxford Centre for Evidence-Based Medicine Levels of Evidence.*
