# Supplementary material for: Operationalizing fear of cancer recurrence care: a modified Delphi study establishing a consensus-based clinical pathway for Danish cancer survivors
Source: Acta Oncol. 2026 Sep 24;65:46444. doi: 10.2340/1651-226X.2026.46444 (PMC13615413; doi:10.2340/1651-226X.2026.46444)
Supplement: Supplementary file 2 [file AO-65-46444-s2.pdf]

Supplementary material has been published as submitted. It has not been copyedited, or typeset by Acta Oncologica

From guideline to clinical practice: A modified Delphi study on operationalizing fear of cancer recurrence care

Supplementary File 2

Detailed Results from Round 1 and Round 3

This supplementary file provides detailed item-level results from Round 1 and Round 3 of the modified Delphi study on the operationalization of a clinical pathway for fear of cancer recurrence (FCR).

Agreement is reported as the percentage of participants selecting “agree” or “strongly agree”, excluding responses marked as “not within my area of expertise”.

Consensus was defined a priori as ≥80% agreement on optimality.

ROUND 1

Item	Statement (English)	Agreement optimality n/N (%)
**Screening**		
**1**	All cancer survivors should be systematically screened for FCR	**65/80 (81.3%)**
**2**	Screening for FCR should be initiated at the time of diagnosis	13/80 (16.3%)
**3**	Screening for FCR should be initiated at the completion of hospital-based treatment (e.g., surgery, systemic therapy or radiotherapy)	57/80 (71.3%)
**4**	Screening for FCR should be conducted prior to a cancer survivor’s follow-up consultation* to support discussion during the consultation	**66/80 (81.5%)**
**5**	Screening for FCR should be repeated after a cancer survivor’s follow-up consultation to assess baseline FCR levels	41/78 (52.6%)
**6**	A brief, validated tool, administered either in written or oral form, should be used to identify cancer survivors with possible FCR	**72/80 (88.9%)**
**7**	A two-step screening approach should be used: (1) a brief (e.g., single-item) screening tool to identify individuals with possible FCR; (2) a longer (e.g., 9-item) tool to assess severity among those scoring above the cut-off	**73/80 (91.3%)**

**8**	Specific personnel should be designated to review the results of FCR screening	45/79 (57.0%)
**9**	All clinical staff should be trained to conduct FCR screening and respond appropriately	53/80 (66.3%)
**Assessment**		
**11**	Assessment should take place prior to the follow-up consultation*, to identify, normalise and support management of increased FCR at this time	40/80 (50.0%)
**12**	Assessment should take place as part of the follow-up consultation	55/79 (69.6%)
**13**	Assessment should take place after the follow-up consultation, as this may trigger increased FCR	30/77 (39.0%)
**14**	Assessment, including discussion of FCR screening results, should take place with all cancer survivors	38/80 (47.5%)
**15**	Assessment, including discussion of FCR screening results, should only take place with cancer survivors reporting moderate or severe FCR	52/80 (65.0%)
**16**	Training is needed to support healthcare professionals in conducting FCR assessment	**72/78 (92.3%)**
**17**	When screening and assessment results indicate severe FCR, the cancer survivor should be evaluated by a psychologist or psychiatrist to confirm the need for specialised FCR treatment	**60/74 (81.1%)**
**Treatment**		
**19**	All cancer survivors should initially be allocated to Step 1 regardless of initial FCR level and subsequently escalated based on re-screening results (stepped-care)	26/72 (36.1%)
**20**	Cancer survivors should be offered an intervention at the treatment level (1, 2 or 3) that matches the severity of their FCR (matched-care)	**59/73 (80.8%)**
**21**	If the cancer survivor does not engage in the offered FCR intervention, alternative interventions within the same level should be discussed. If none are appropriate, less intensive interventions from the lower level may be considered	52/72 (72.2%)
**22**	Treatment offered to cancer survivors with FCR should take into account FCR severity as well as patient preferences and ability to engage in self-help versus clinician-delivered interventions	**73/77 (94.8%)**
**23**	Re-screening for FCR during routine follow-up consultations is an appropriate way to monitor changes and guide further assessment and treatment if needed	**63/77 (81.8%)**
**24**	If cancer survivors report normal or mild FCR at re-screening, they should continue to be screened and be offered information on self-management and available support options in case of worsening FCR	47/75 (62.7%)

**25**	Booster sessions should be offered to cancer survivors with persistent treatment-requiring FCR at re-screening	44/65 (67.7%)
**26**	Patients should be re-screened 6 months after intervention to assess long-term effects	**60/73 (82.2%)**

ROUND 3

**Item**	**Statement (English)**	**Agreement optimality n/N (%)**
**Screening**		
**1a**	Screening for FCR should be initiated at the completion of hospital-based treatment (e.g., surgery, systemic therapy or radiotherapy)	49/68 (72%)
**1b**	Screening for FCR should be initiated at the transition from treatment (e.g., surgery, systemic therapy or radiotherapy) to follow-up	**57/69 (83%)**
**1c**	Screening for FCR should be conducted continuously during prolonged hospital-based treatment and at minimum at the completion of treatment	51/70 (73%)
**Pref 1**	Preference: Version 1a vs 1b vs 1c	**1b preferred (44%)**
**2**	Results from FCR screening should be included in communication to the general practitioner	**69/70 (99%)**
**3**	Screening for FCR should be conducted as part of follow-up consultations in general practice	**64/70 (91%)**
**4a**	Screening for FCR should be repeated after a cancer survivor's follow-up consultation to assess baseline FCR levels	27/70 (39%)
**4b**	Screening for FCR should not be repeated after a cancer survivor's follow-up consultation to assess baseline FCR levels	30/69 (43%)
**Pref 4**	Preference: Version 4a vs 4b	4b preferred (56%)
**5a**	Specific personnel should be designated to review the results of FCR screening	36/68 (53%)
**5b**	All clinical staff should be trained to conduct FCR screening and respond appropriately	40/70 (57%)
**5c**	All clinical staff should have knowledge of FCR screening and be able to respond appropriately	**63/70 (90%)**
**5d**	Clear workflows for FCR screening should be established, and relevant personnel should be trained to conduct screening, interpret results, and determine indication for assessment	**68/70 (97%)**
**5e**	The physician/nurse should review FCR screening results prior to the follow-up consultation	**68/70 (97%)**
**Pref 5**	Preference: Version 5a–5e	**5d preferred (66%)**

**Assessment**		
**6**	Assessment should take place prior to the follow-up consultation to identify, normalise and support management of increased FCR	27/68 (40%)
**7**	Assessment should take place as part of the follow-up consultation	55/71 (77%)
**8a**	Assessment, including discussion of FCR screening results, should only take place with cancer survivors reporting moderate or severe FCR	52/67 (78%)
**8b**	Assessment should take place if the patient reports moderate to severe FCR on screening or expresses a need (verbally or non-verbally) for assessment	**66/70 (94%)**
**Pref 8**	Preference: Version 8a vs 8b	**8b preferred (92%)**
**Treatment**		
**9a**	If the cancer survivor does not engage in the offered FCR intervention, alternative interventions within the same level should be discussed. If none are appropriate, less intensive interventions from the lower level may be considered	55/69 (80%)
**10a**	If cancer survivors report normal or mild FCR at re-screening, they should continue to be screened and be offered information on self-management and support options in case of worsening FCR	41/70 (59%)
**11a**	Booster sessions should be offered to cancer survivors with persistent treatment-requiring FCR at re-screening	52/67 (78%)
**9b–11b**	If the cancer survivor does not improve following the offered FCR intervention, or continues to report treatment-requiring FCR at re-screening, care should be re-evaluated in collaboration with the cancer survivor to determine further treatment	**70/71 (99%)**
**Pref 9–11**	Preference: Version 9a–11a vs 9b–11b	**9b–11b preferred (82%)**
